# Diverticular disease hospital cost impact analysis: evaluation of testings and surgical procedures in inpatient and outpatient admissions

**DOI:** 10.1186/1471-2482-12-S1-S3

**Published:** 2012-11-15

**Authors:** Giovanni Aprea, Antonio Giugliano, Alfonso Canfora, Fabrizio Cardin, Antonio Ferronetti, Francesco Guida, Antonio Braun, Melania Battaglini Ciciriello, Federica Tovecci, Giovanni Mastrobuoni, Bruno Amato

**Affiliations:** 1 Department of General, Geriatric, Oncologic Surgery and Advanced Technologies, University “Federico II” of Naples. Via Pansini, 5 - 80131 – Naples, Italy; 2 Department of Surgical and Gastroenterological Sciences, Padova University Hospital, Italy, Via Giustiniani n.2, 35126 Padova, Italy

## Abstract

**Background:**

Diverticular Disease (DD) is a common condition in Italy and in other western countries. There is not much data concerning DD’s impact on budget and activity in hospitals.

**Methods:**

The aim is to detect the clinical workload and the financial impact of diverticular disease in hospitals.

Retrospective observational study of all patients treated for diverticular disease during the period of seven years in AOU Federico II. Analysis of inpatient and outpatient investigations, treatment, hospitalization and financial refunds.

**Results:**

A total of 738 patients were treated and 840 hospital discharge records were registered. There were a total number of 4101 hospitalization days and 753 outpatient accesses. The investigations generated were 416 endoscopies, 197 abdominal CT scans, 177 abdominal ultrasound scans, 109 X-rays tests. A total of 193 surgical operations were performed. The total cost of this activity was € 1.656.802 or 0.2% of the total budget of the hospital. € 1.346.218, were attributable to the department of general surgery, 0.9% of the department’s budget .

**Conclusions:**

The limited impact of diverticular disease on the budget and activity of AOU Federico II of Naples is mainly due to the absence of an emergency department.

## Background

Diverticular Disease (DD) is a common condition in Italy and in other western countries [1-3]. The prevalence of diverticulosis in the general population is believed to be around 27% and increases with age [4]. Nevertheless we could not find in literature many recorded data on the impact of DD on a health system’s costs and activity. Indeed there is only one clinical study in literature evaluating this aspect of DD [5]. According to this study, performed at the James Cook University Hospital in UK, the DD represented 5.3% of the total budget of the department of General Surgery. The aims of our study were to record the clinical workload and calculate the financial cost generated by DD in A.O.U Federico II of Naples.

## Methods

A list of all hospital discharge records (SDO), which were coded as having Diverticular Disease as the primary condition (corresponding to icd-9cm: 562.10; 562.11; 562.12; 562.13)during a period of seven years between 2004 and 2010, was obtained from the Health Department of A.O.U Federico II.

This was the result of a computer search in the archive of our Health Department. The hospital discharge records (SDO) were divided into inpatient admissions and outpatient accesses. The SDO were also divided by hospital department.

In order to simplify this subdivision five macro-groups of departments were created: General Surgery, Gastroenterology, Internal Medicine, Geriatrics, and Other.

The examined parameters included inpatient admissions, outpatient accesses, hospitalization days, diagnostic tests, surgery and economic refunds.

## Results and discussion

A total of 738 patients were treated in the period between January 2004 and December 2010. There were 840 hospital discharge records corresponding to these patients: of these 427 were inpatient and 413 were for outpatient care.

Diverticular disease accounted for 0.19% of the inpatient admissions and 0.13% of the outpatient. These patients produced a total of 4101 bed-days (corresponding to 0.29% of the total count of hospital bed-days) and 753 day-care access (corresponding to 0.12% of the total number of hospital outpatient accesses).

The data analysis shows a reduction in the flow of admissions in 2008, with a reduction of 24% compared to 2004, and in 2010, with a reduction of 44% compared to 2004. This decrease was contextual to a reduction in the number of admissions throughout the hospital for all diseases, therefore, does not cause statistically significant changes in percentage terms.

**Figure 1:**
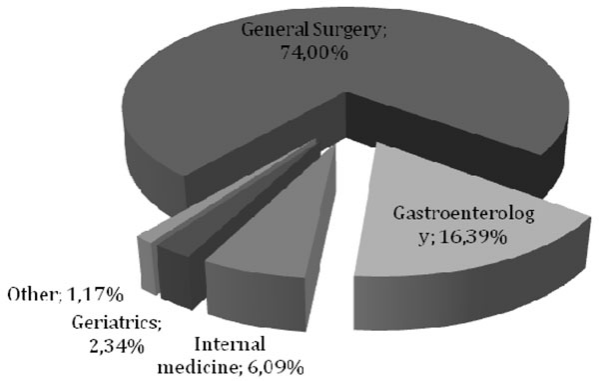
Diverticular disease’s regular admission distribution in AOU Federico II di Napoli in the period 2004-2010.

**Figure 2:**
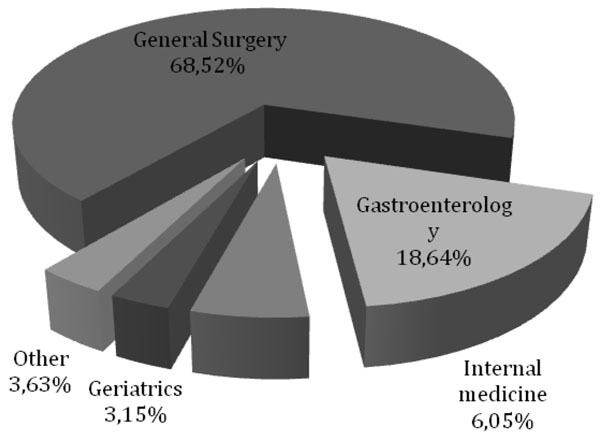
Diverticular disease’s day hospital admission distribution in AOU Federico II di Napoli in the period 2004-2010.

The admissions for diverticular disease were unevenly distributed among the departments. In fact 74% of inpatient admissions were recorded in the department of general surgery and 16.39% in the department of gastroenterology (Fig. 1). Similar results were also observed for admissions to outpatient care: 68.52% of the outpatient admissions were recorded in general surgery and 18.64% in gastroenterology (Fig.2).

**Figure 3:**
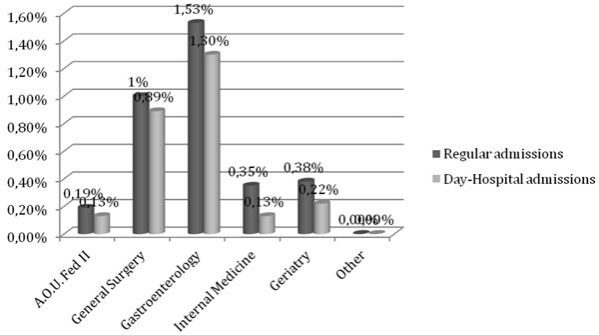
Period 2004-2010, data grouped for department: in dark ratio between DD’s regular accesses and overall regular accesses; in light ratio between DD’s day hospitals and overall day hospitals.

Therefore diverticular disease had a relative weight on admissions in these two units greater than that recorded for the whole hospital. In fact it represented 1% of inpatient admissions and 0.89% of outpatient general surgery, and it represented 1.53% of inpatient admissions and 1.30% of the outpatient gastroenterology (Fig.3).

**Figure 4:**
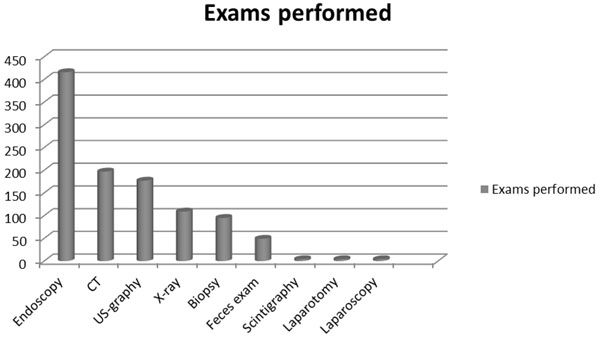
Examinations performed for DD in AOU Federico II di Napoli in the period 2004-2010.

The investigations used (blood tests excluded) were: 416 endoscopies, 197 abdominal CT scans, 177 abdominal ultrasound scans, 109 X-rays tests, 95 biopsies, 49 fecal examination, 4 scintigraphy scans, 4 laparotomies and 4 laparoscopies (Fig.4).

A total of 193 surgical operations were performed. These consisted of 68 sigmoid colectomies, 58 left hemicolectomies and only thirteen colostomies were performed. The ratio between the number of surgical procedures and inpatient admissions in the department of general surgery was about 60% (Tab.1).

**Table 1:** Surgical interventions performed for diverticular disease in the period 2004-2010

Intervention	Number	Percentage
Sigmoidectomy	68	35.2%

Left Hemicolectomy	58	30.1%

Total colectomy	9	4.7%

Other partial colectomies	34	17.6%

Artificial orifice interventions	20	10.4%

Intestinal anastomosis	4	2%

**Total**	193	100%

The overall cost of DD was € 1.656.802. This sum represented 0.2% of the total budget of the hospital. The major part of the cost, € 1.346.218, was attributable to the department of general surgery, representing 0.9% of the department’s budget, while € 182.124 were attributable to the department of gastroenterology, representing 1.02% of the department’s budget .

Our data show a very different setting in comparison to James Cook University Hospital. In fact, the DD is only 0.9% of the budget of the department of general surgery of A.O.U Federico II of Naples and 0.2% of the budget of the entire hospital.

We believe that the main cause of this difference is the absence of an emergency department in A.O.U Federico II. The DD is considered one of the most common causes of hospitalization in a surgical ward and the presence of diverticulitis and its complications are the leading cause of hospitalization for diverticular disease [6-9]. This is confirmed by the case studies reported in literature, according to which about 90% of surgical procedures for DD is performed in emergency conditions [10,11].

Our hypothesis is supported by data on diagnostic and surgical procedures.

First of all we observe that the endoscopies, which are contraindicated in presence of an acute inflammation [2,12,13], represent about 40% of diagnostic tests, while CT scans only 18%. This first finding suggests that our hospital admits mainly patients with a non-acute diverticular disease, in a phase between attacks [14].

Secondly our data shows that a total of 159 partial colectomies were performed, while only 13 colostomies were performed. As it is well known, the creation of a colostomy is indicated in presence of complications of diverticulitis, while without an emergency situation it is preferable to perform a resection with primary anastomosis [2,11]. Even this data confirms the presence of a high number of patients in a non-acute phase at the time of admission.

**Figure 5:**
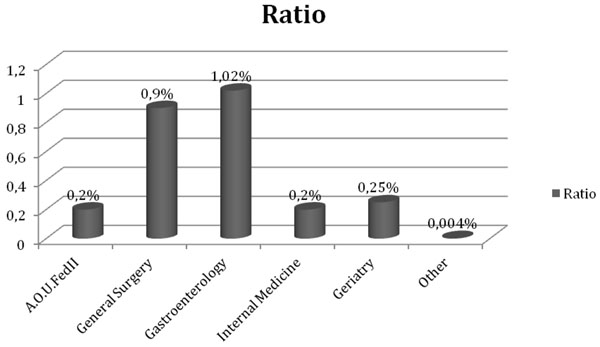
Ratio between DD’s economic refund and overall refund for singular department in the period 2004-2010.

Moreover, in our hospital the ratio between interventions and inpatient admissions in general surgery was 60% (Fig.5), while at James Cook University Hospital only 13% of patients underwent surgery [5]. This finding gives even more significance to the difference between the impact of DD on the budget in our general surgery department and James Cook University Hospital’s.

## Conclusions

In conclusion, our data suggest that the marked difference between the results of our clinical study and the research conducted at the UK hospital is due to the absence of an emergency department in our hospital. However, this hypothesis can be confirmed only by direct comparison with another hospital of the same region providing an emergency department.

## List of abbreviations

A.O.U.: Azienda ospedaliera universitaria (University hospital); DD: Diverticular disease; SDO: Scheda dimissione ospedaliera (Hospital discharge record)

## Competing interests

The authors declare that they have no competing interests.

## Authors' contributions

GA, BA: conception and design, interpretation of data, given final approval of the version to be published; AG, AC, AF, FG, AB, MBC,FT,GM: acquisition of data, drafting the manuscript, given final approval of the version to be published; FC: acquisition of data, drafting the manuscript, given final approval of the version to be published.
